# ICSI with Testicular Sperm in Non-Azoospermic Men: Expert Opinion and Practical Algorithm

**DOI:** 10.1590/S1677-5538.IBJU.2025.9916

**Published:** 2025-08-30

**Authors:** Sandro C. Esteves

**Affiliations:** 1 ANDROFERT Clínica de Andrologia e Reprodução Humana Campinas SP Brasil ANDROFERT, Clínica de Andrologia e Reprodução Humana, Campinas, SP, Brasil; 2 Universidade Estadual de Campinas Faculdade de Ciências Médicas Departamento de Cirurgia (Disciplina de Urologia) Campinas SP Brasil Departamento de Cirurgia (Disciplina de Urologia), Faculdade de Ciências Médicas, Universidade Estadual de Campinas (UNICAMP), Campinas, SP, Brasil; 3 Aarhus University Department of Clinical Medicine Aarhus Denmark Department of Clinical Medicine, Aarhus University, Aarhus, Denmark

## INTRODUCTION

Sperm retrieval (SR) was pioneered for obstructive and non-obstructive azoospermia, with ICSI required because epididymal or testicular sperm are not competent for fertilization via conventional IVF. As ICSI expanded globally, clinicians recognized that poor ejaculate quality can impair ICSI outcomes, and that testicular sperm—being shielded from epididymal transit and ejaculatory oxidative stress—may confer a genomic advantage in selected non-azoospermic men. Concurrently, assays quantifying sperm DNA fragmentation (SDF) clarified a mechanistic link between oxidative damage and reproductive failure. This Expert Opinion integrates mechanistic rationale, center-derived prevalence data on high SDF, evidence from meta-analyses and cohorts, laboratory adjuncts, and a pragmatic algorithm for using testicular in preference over ejaculated sperm for ICSI (Testi-ICSI).

### Biological Rationale

Human spermatozoa are uniquely vulnerable to reactive oxygen species (ROS) due to limited cytoplasm, abundant polyunsaturated fatty acids in membranes, and highly condensed chromatin with residual nicks. Clinically relevant ROS sources include varicocele, genital tract infection, febrile illness, environmental toxins, metabolic disease, smoking, and advanced paternal age (1, 2). ROS attack lipids and DNA, producing oxidative adducts such as 8-oxo-deoxyguanosine (8-oxo-dG) (3, 4). The OGG1 glycosylase removes 8-oxo-dG, creating an abasic site that, in the absence of downstream base-excision repair machinery—which mature sperm lack—renders DNA susceptible to strand breaks (4, 5).

Following fertilization, the oocyte's repair systems must correct paternal lesions before syngamy. When the paternal lesion burden is high or the oocyte's repair reserve is diminished (such as in advanced maternal age and diminished ovarian reserve), incomplete repair may manifest as delayed paternal DNA replication, impaired embryo development, implantation failure, miscarriage, and potentially intergenerational effects (4, 6).

Testicular sperm typically carry far less DNA damage than sperm exposed to the epididymis, vas deferens, and ejaculate (4, 7-13). The key factor appears to be the minimization of oxidative stress encountered during transit and after ejaculation (14, 15). This biology and collective data provide a mechanistic basis for preferring testicular sperm—less exposed to post-testicular oxidative stress—in specific clinical contexts.

### SDF and ART Outcomes: Meta-Analytic Signals

Systematic reviews and meta-analyses have examined SDF in relation to reproductive outcomes across natural conception, IUI, IVF, and ICSI. A synthesis of at least twelve meta-analyses since 2006 indicates pregnancy-rate findings vary by assay and threshold (16). Two analyses reported no effect of SDF on pregnancy, eight showed a clear adverse association for conventional IVF but not for ICSI, and two indicated an adverse effect in both IVF and ICSI (16). Heterogeneity arises from differences in SDF assays (e.g., SCD, TUNEL, SCSA, Comet), thresholds, patient selection, and denominators (per embryo transfer vs per initiated cycle).

Despite divergent pregnancy signals, an elevated risk of miscarriage after IVF/ICSI in high-SDF cohorts is a consistent finding across summaries (16). Signals linking SDF to impaired embryo development and possibly higher aneuploidy reinforce the biological plausibility of reducing the paternal DNA lesion burden in selected couples (17, 18). Clinically, outcomes reflect the balance between the magnitude of sperm DNA damage and the capacity of the oocyte to repair paternal lesions—an equilibrium often unfavorable in the presence of advanced maternal age or diminished ovarian reserve (4).

### Who Benefits from Testi-ICSI Among Non-Azoospermic Men?

The most coherent signal emerges in couples with elevated ejaculate SDF (9-11, 14). In cohorts with failed IVF/ICSI and high SDF, switching to Testi-ICSI was associated with higher pregnancy rates (19-22). Similar trends are reported in ART-naïve couples selected for high SDF (Table-1). These findings are echoed by meta-analyses pooling comparative and case-series data (10, 23-25).

**Table 1:** Comparative trials using Testi-ICSI in high-SDF settings.

Study	Male population	High SDF criterion/ Prior ART?	N (couples/cycles)	Design	Primary outcome	Key findings
Esteves et al., 2015 (12)	Idiopathic oligozoospermia	SCD>30%; ART-naïve	172 couples	Prospective cohort	Live birth rate	LBR ↑ 46.7 (T) vs. 26.4 (E), p=0.007; NNT≈5; miscarriage ↓
Bradley et al., 2016 (24)	Mainly oligozoospermic	SCIT>29%; ART-naïve	228 cycles	Retrospective cohort	Live birth rate	LBR ↑ 49.8 (T) vs. 24.2 (E), p<0.05
Pabuccu et al., 2017 (21)	Normo-zoospermic	TUNEL>30% Failed ART	71 couples	Retrospective cohort	Ongoing pregnancy rate	OPR ↑ 38.7 (T) vs. 15.0 (E), p=0.02
Zhang et al., 2018 (25)	Mixed (oligo- and normo-zoospermic)	Not specified	102 couples	Prospective cohort	Live birth rate	LBR ↑ 36.0 (T) vs. 9.8 (E), p=0.001
Herrero et al. 2019 (20)	Not specified	SCSA>25% or TUNEL>36%; failed ART	145 couples	Retrospective cohort	Cumulative live birth rate	CLBR↑ SCSA: 21.7 (T) vs. 9.1 (E), p<0.01; TUNEL: 20.0 (T) vs. 0.0 (E), p<0.02
Alharbi et al. 2020 (19)	Not specified	SCSA>15%; failed ART	100 couples	Retrospective cohort	Clinical pregnancy rate	CPR ≈ 36.4 (T) vs. 30.0 (E), p=0.59
Benchaib et al. 2024 (22)	Not specified	TUNEL>15%; failed ART	126 couples	Retrospective cohort; propensity score matching	Cumulative live birth rate	CLBR ↑ (trend) 25.4 (T) vs. 6.3 (E), p=0.06

OPR = ongoing pregnancy rate; SCD = sperm chromatin dispersion test; SCIT = sperm chromatin integrity test, a variation of sperm chromatin structure assay (SCSA); TUNEL = terminal deoxyribonucleotide transferase–mediated dUTP nick-end labeling assay; T = testicular sperm; E = ejaculated sperm

In our prospective cohort of approximately 170 couples with idiopathic oligozoospermia and elevated SDF, switching to testicular-sperm ICSI reduced miscarriage and increased live birth versus ejaculated-sperm ICSI (12). The number-needed-to-treat was about five to achieve one additional delivery, underscoring a clinically meaningful effect size in this phenotype (12). Paired assessments demonstrated ∼80% lower SDF in testicular versus ejaculated sperm from the same men, consistent with minimized post-testicular oxidative exposure (12).

Notably, a large cohort using a semen count threshold (e.g., TMSC <25 million) to allocate men to Testi-ICSI showed no benefit, emphasizing that total motile count alone is not an appropriate selection criterion (26). Instead, the indication should be driven by post-optimization SDF status and clinical context (e.g., prior implantation failure, miscarriage, or poor embryo development).

### What the Guidelines Say

The 2024 AUA/ASRM male infertility update acknowledges that clinicians may consider testicular sperm in non-azoospermic men with elevated SDF—a clinical principle reflecting limited but convergent evidence (27). Dedicated SDF guidance provides practical recommendations on testing and management and is congruent with the findings presented here (1, 2, 28, 29).

### SDF Assays and Thresholds: Practicalities and Pitfalls

Clinical practice hinges on assay choice, timing, and interpretation. The most widely adopted assays—SCD, TUNEL, SCSA, and Comet—interrogate overlapping but non-identical lesion spectra. SCD reflects chromatin dispersal after denaturation; TUNEL labels DNA breaks; SCSA measures susceptibility to acid-induced denaturation by flow cytometry; Comet quantifies strand migration at the single-cell level (2). Assay variability is exacerbated by abstinence period, fever or systemic illness in the prior three months, and pre-analytic handling (temperature, time to processing) (2). Quality control is critical: duplicate slides, positive controls, and laboratory-specific calibration should be standard. In this framework, a pragmatic threshold of 20% marks the onset of pathological SDF for many assays, whereas ≥30% denotes a zone where adverse reproductive signals strengthen (2, 30). Importantly, thresholds are assay-specific; clinicians should avoid direct transposition of cut-offs across platforms and, instead, anchor decisions to validated local performance data (2, 4, 30).

SDF should be measured after optimization of reversible factors to avoid misclassification. Varicocele repair, treatment of genital tract infection, smoking cessation, weight management, and correction of clinical hypogonadism may meaningfully reduce SDF in a subset of men (1, 2, 4, 14, 28, 31-36). A repeat SDF test 8–12 weeks after intervention aligns with one spermatogenic cycle. Where resources allow, pairing a general SDF assay with an oxidative-damage-focused readout (e.g., 8-oxo-dG) can refine counseling about mechanism and prognosis.

### Center Experience: Prevalence of Elevated SDF

In our center, where we routinely perform SDF screening as part of the basic semen analysis, >50% of our patients show SDF >20% (our pathological threshold for the SCD test), and >25% have SDF ≥30%, where adverse effects on reproductive outcomes become more evident (4, 30).

### Cryptozoospermia: Feasibility, Focality, and Surgical Nuances

In cryptozoospermia, the existing evidence from comparative trials is mixed, although most studies suggest that Testi-ICSI may improve the likelihood of achieving good-quality embryos, implantation, and pregnancy (Table-2) (37-39). However, the number and quality of trials, and the number of patients studied, are lower than in the SDF scenario. Meta-analyses pooling comparative and case-series data suggest superiority of Testi-ICSI in this setting (40, 41). Moreover, SDF measurement is often impractical due to extremely low sperm counts. After optimizing reversible male factors, an initial attempt using ejaculated sperm can be reasonable when technically feasible. If ART fails or when embryo development is suboptimal, Testi-ICSI becomes a rational escalation.

**Table 2:** Comparative trials using Testi-ICSI in cryptozoospermia settings.

Study	Population/ Prior ART?	N (couples/cycles)	Design	Primary outcome	Key findings
Ketabchi et al., 2016 (38)	Not specified	73 couples	Prospective cohort	Clinical pregnancy rate	CPR ↑ 57.1 (T) vs. 31.6 (E), p<0.001
Cui et al., 2017 (37)	Not specified	285 couples	Retrospective cohort	Live birth rate	LBR ↑ 44.0 (T) vs. 27.1 (E), p=0.03
Yu et al., 2019 (39)	Not specified	35 couples	Prospective cohort	Live birth rate	52.9 (T) vs. 44.4 (E) in men <35 years-old (NS); 42.9 (T) *vs.* 0.0 (E) in men ≥35 years-old (p not reported)

CPR = clinical pregnancy rate; T = testicular sperm; E = ejaculated sperm; NS = not statistically significant

Given the focal nature of spermatogenesis in this context, micro-TESE often yields higher retrieval success than blind needle aspiration (42); however, conventional TESE may succeed in selected cases. When proceeding, synchronizing sperm retrieval with oocyte pick-up on the same day streamlines laboratory logistics. Whether fresh testicular sperm outperforms frozen remains unsettled and may depend on center expertise and logistics.

### Laboratory Adjuncts: Microfluidics in Context

Microfluidic sperm selection seeks to exploit laminar-flow behaviors to enrich for motile, morphologically intact sperm with fewer DNA lesions. A recent systematic review and meta-analysis of 39 studies (9 RCTs) comparing microfluidics with conventional preparation (swim-up/density gradients) reported: SDF reduction ≈10 points (MD−9.98; p<0.001), pregnancy per-embryo transfer gains (fertilization/MII OR 1.22, implantation OR 4.51, clinical pregnancy OR 1.73, ongoing pregnancy OR 1.99, a live birth per first cycle OR 1.59, and per all embryo transfers OR 1.65) (43). However, no significant differences were observed in embryo euploidy, biochemical pregnancy, miscarriage (per cycle or pregnancy), live birth per first embryo transfer, or live birth per concluded cycle.

While per-transfer improvements suggest enhanced embryo competence among selected sperm; however, denominators matter. Gains per embryo transfer may not translate into consistent gains per first embryo transfer or per concluded cycle. Moreover, in the meta-analysis mentioned above, none of the existing RCTs target high-SDF men—precisely the group where selection technologies might matter most—and none compared microfluidics head-to-head with testicular sperm (43). Accordingly, we position microfluidics as an adjunct in intermediate SDF at first ART, reserving Testi-ICSI for persistent high SDF or after failed ART. At present, evidence does not support routine adoption of microfluidics to unselected patients undergoing sperm preparation for IVF/ICSI, and cost-effectiveness remains to be determined. In practice, microfluidics can be considered particularly for intermediate SDF (20-29%) at a first IVF/ICSI attempt—while Testi-ICSI remains the stronger option in persistently high SDF (≥30%) or after ART failure.

### Practical Algorithm

**Figure 1:**
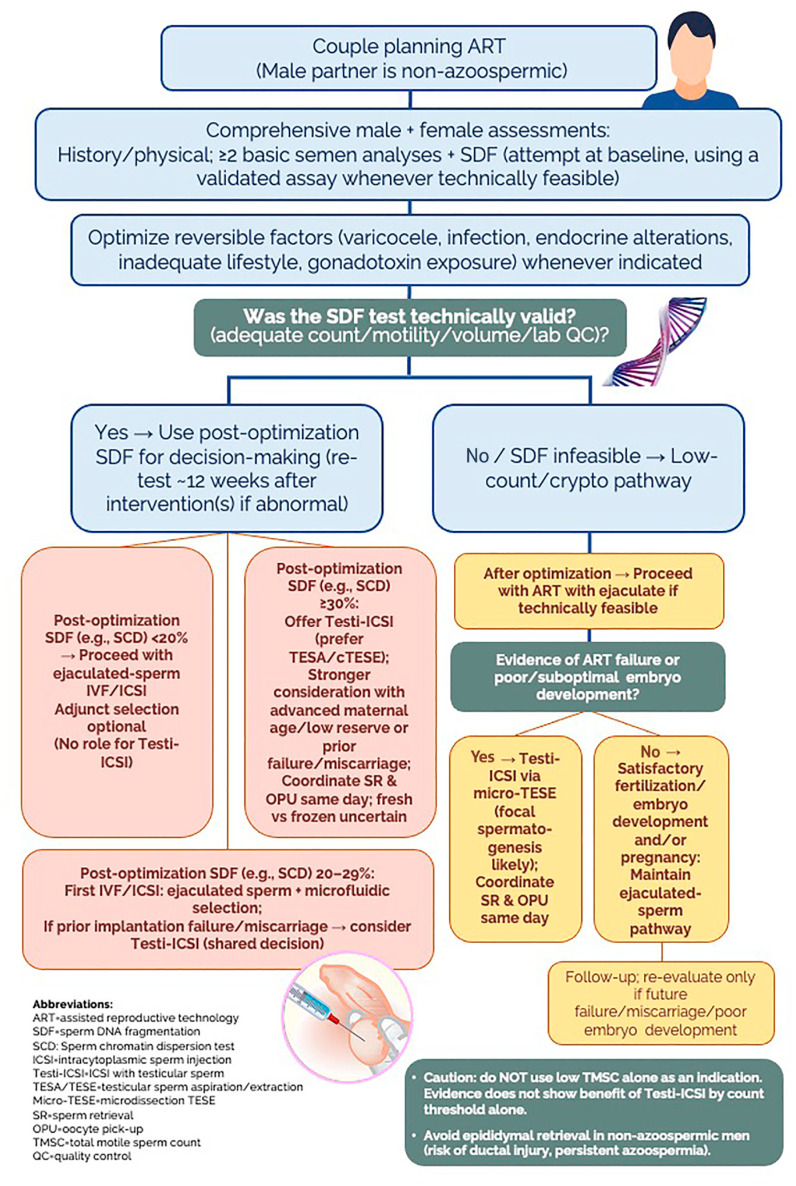
Algorithm for selecting ICSI with testicular sperm (Testi-ICSI) in non-azoospermic men.

Figure-1 presents an algorithm that anchors decision-making to comprehensive SDF testing, correction of reversible male factors, and phenotype-guided insemination strategy.

Start with comprehensive male and female assessment (history, physical examination, basic semen analyses, and SDF testing with a validated assay whenever technically feasible); correct reversible male factors (varicocele, infection, endocrine disorders, toxic exposures, lifestyle), and reassess; consider antioxidants case-by-case. If SDF can be reliably measured in ejaculate, test after optimization. For SDF <20% (e.g., by SCD): proceed with ejaculated-sperm IVF/ICSI; adjunct selection is optional; Testi-ICSI is not indicated. For SDF 20–29%: at first IVF/ICSI consider microfluidic selection with ejaculated sperm; after prior implantation failure or miscarriage, Testi-ICSI may be considered following shared decision-making. For SDF ≥30%: offer Testi-ICSI (often TESA or TESE). Give stronger consideration with advanced maternal age or low ovarian reserve, and after prior implantation failure or miscarriage. Coordinate sperm retrieval and oocyte pick-up on the same day (fresh vs frozen unresolved). When SDF is infeasible/invalid (low count/crypto), attempt ART with ejaculate if possible; escalate to Testi-ICSI via micro-TESE only upon ART failure or suboptimal embryo development; otherwise maintain the ejaculated-sperm pathway and follow up. Caution: low total motile sperm count alone is not an indication; epididymal retrieval should be avoided in non-azoospermic men. Thresholds are assay specific.

After optimization:

- SDF <20% supports proceeding with ejaculated-sperm IVF/ICSI;
- SDF 20–29% favors ejaculated sperm with microfluidic selection at the first IVF/ICSI, while prior implantation failure or miscarriage can justify Testi-ICSI after shared decision-making;
- SDF ≥30% supports offering Testi-ICSI (often via TESA/TESE; micro-TESE in focal cases), particularly when oocyte repair reserve is likely constrained by age or low ovarian reserve.

For cryptozoospermia, after optimization, an initial ejaculated-sperm attempt is reasonable; ART failure or poor embryo development should trigger Testi-ICSI consideration via micro-TESE. Safeguards include avoiding epididymal retrieval in non-azoospermic men and avoiding low TMSC alone as an indication.

### Surgical Approaches: TESA, TESE, and micro-TESE

TESA (percutaneous testicular sperm aspiration), TESE (conventional testicular sperm extraction by open biopsy), and micro-TESE (microsurgical testicular sperm extraction) are complementary techniques (14, 33, 44, 45). In non-azoospermic men with diffuse spermatogenesis, TESA and TESE are sufficient for Testi-ICSI in the high-SDF context, minimizing invasiveness and recovery time (12, 14). In cryptozoospermia or when prior needle aspiration fails, micro-TESE maximizes retrieval by targeting larger, opaque tubules under magnification, consistent with focal spermatogenesis (14, 42, 46).

Complication rates are low in experienced hands: hematoma and infection are uncommon; transient pain is the most frequent complaint (14). Endocrine sequelae are rare in non-azoospermic men, especially with small-volume, strategically sampled tissue (14). We avoid epididymal approaches in non-azoospermic men to prevent ductal injury and the risk of persistent obstructive azoospermia. Additionally, we do not use low TMSC alone as an indication. Mechanistically, choosing testicular sperm seeks to limit the cumulative oxidative insults encountered during epididymal transit and ejaculation.

Synchronizing sperm retrieval with oocyte pick-up simplifies laboratory coordination and optimizes outcomes (47). Nevertheless, centers should maintain validated cryopreservation protocols for testicular sperm, including rapid identification and isolation of motile sperm, minimizing red blood cell contamination, and using small-volume straws compatible with the micro-injection workflow. Where fresh retrieval is planned, a backup semen option (fresh or frozen) should be considered to mitigate the risk of unexpected retrieval failure or poor sperm quality on the day.

### Counseling, Risks, and Logistics

Shared decision-making should cover the rationale for Testi-ICSI, expected benefits, and uncertainties. Counseling should explicitly address:

- expected procedural recovery for TESE/micro-TESE (review anesthesia and surgical risks such as hematoma, infection, pain);
- likelihood of retrieval success by phenotype (retrieval failure risk in cryptozoospermia);
- laboratory coordination (same-day SR and OPU when feasible);
- options if retrieval is unexpectedly poor (cryopreserved backup, oocyte vitrification);
- expected benefits (e.g., miscarriage reduction in high-SDF cohorts);
- current evidence uncertainties (offspring health data and the comparative outcomes of fresh versus frozen testicular sperm).

Centers should standardize internal denominators (per MII injected, per ET, per initiated cycle) for transparent reporting. Embryo-transfer policy should reflect the couple's prognosis, embryo quality, and local regulations, with preferential single-embryo transfer to reduce multiple gestation risk. Centers should also record whether embryos derive from ejaculated versus testicular sperm to facilitate internal audits and research.

Furthermore, clinicians should document counseling about guideline status (clinical principle rather than strong recommendation) and align expectations around alternative or adjunct strategies such as microfluidics. In men with treatable causes (e.g., varicocele, hypogonadism), the underlying condition should be addressed first, where feasible, to potentially lower SDF and improve ejaculate quality.

### Cost-Effectiveness and Quality Indicators/Auditing

Robust cost-effectiveness analyses are scarce. Testi-ICSI adds operating room time, anesthesia, and potential complications, whereas microfluidics adds disposables and bench time. Economic value will hinge on time-to-pregnancy, cumulative live birth per started cycle, and avoidance of miscarriage-related costs. Programs should capture direct and indirect costs and consider pragmatic sequencing (e.g., microfluidics for SDF 20–29% at first ART, reserving Testi-ICSI for persistent SDF ≥30% or failed ART).

As far as quality indicators and auditing are concerned (47), a simple dashboard can track:

- proportion of ART patients undergoing SDF testing;
- post-optimization SDF distribution;
- uptake of microfluidics and Testi-ICSI by indication;
- retrieval success rates (TESA/TESE/micro-TESE) and complications;
- fertilization, blastulation, implantation, miscarriage, and live birth stratified by sperm source;
- time-to-pregnancy and cumulative live birth.

Regular feedback loops align the algorithm with local performance and support shared decision-making.

### Limitations, Knowledge Gaps, and Research Priorities

Much of the published evidence derives from observational cohorts subject to selection and residual confounding. Many studies rely on per-transfer denominators, which can inflate apparent effect sizes if more embryos are generated in one arm. Assay heterogeneity complicates threshold generalization; centers should validate their own performance characteristics and avoid cross-platform cut-off transposition. In Testi-ICSI cohorts, concomitant interventions (e.g., varicocele repair, antioxidant use) are not always balanced or reported. Finally, outcome reporting rarely stratifies by maternal age or oocyte source, variables that directly modulate the oocyte repair reserve. These caveats argue for cautious interpretation and reinforce the need for transparent local audits.

Implementation should:

- standardize SDF testing after optimization;
- embed a clear consent pathway covering benefits, risks, and uncertainties;
- align surgical and laboratory teams on same-day logistics;
- report outcomes using multiple denominators (per MII, per ET, per initiated cycle) with explicit indication categories (high-SDF, cryptozoospermia) to enable fair benchmarking and quality improvement.

Research priorities include phenotype-specific trials (high-SDF men) comparing Testi-ICSI vs optimized ejaculated-sperm ICSI (± microfluidics), prospective cryptozoospermia cohorts with live-birth/offspring outcomes, assay- and threshold-specific performance metrics, head-to-head microfluidics vs Testi-ICSI, and cost-effectiveness analyses.

## CONCLUSIONS

Testi-ICSI is not a universal solution for non-azoospermic male factor. Yet in the right patient—particularly with persistently high SDF despite optimization or with cryptozoospermia after failed ART—it can be a decisive intervention. Mechanistic plausibility, meta-analytic signals (especially for miscarriage), cohort data, and cautious guideline endorsement together support its selective use. A disciplined, algorithm-driven pathway that foregrounds comprehensive andrology, validated SDF testing, judicious use of microfluidics, and careful surgical technique is the safest way to bring this option to the couples most likely to benefit.

### Key Points

- Testicular sperm may bypass post-testicular oxidative damage, reducing paternal DNA lesion burden in selected men.
- Center data: routine SDF screening reveals a high frequency of pathological SDF, supporting targeted pathways.
- Testi-ICSI signal is strongest in high-SDF cohorts, particularly after failed ART; low TMSC alone is not an indication.
- Microfluidics is a useful adjunct in intermediate SDF (20-29%) but lacks consistent live-birth benefit across denominators; no head-to-head trials exist vs testicular sperm.
- The algorithm standardizes selection, counseling, and logistics while acknowledging evidence gaps.

## Data Availability

Not applicable
